# The YvfTU Two-component System is involved in *plcR *expression in *Bacillus cereus*

**DOI:** 10.1186/1471-2180-8-183

**Published:** 2008-10-16

**Authors:** Julien Brillard, Kim Susanna, Caroline Michaud, Claire Dargaignaratz, Michel Gohar, Christina Nielsen-Leroux, Nalini Ramarao, Anne-Brit Kolstø, Christophe Nguyen-the, Didier Lereclus, Véronique Broussolle

**Affiliations:** 1 UMR408, Sécurité et Qualité des Produits d'Origine Végétale, INRA, Université d'Avignon, F-84000 Avignon, France; 2 Department of Pharmaceutical Biosciences, School of Pharmacy, University of Oslo, Norway; 3 INRA, Unité de Génétique Microbienne et Environnement, La Minière, Guyancourt, France; 4 Department of Molecular Genetics, University of Groningen, the Netherlands

## Abstract

**Background:**

Most extracellular virulence factors produced by *Bacillus cereus *are regulated by the pleiotropic transcriptional activator PlcR. Among strains belonging to the *B. cereus *group, the *plcR *gene is always located in the vicinity of genes encoding the YvfTU two-component system. The putative role of YvfTU in the expression of the PlcR regulon was therefore investigated.

**Results:**

Expression of the *plcR *gene was monitored using a transcriptional fusion with a *lacZ *reporter gene in a *yvfTU *mutant and in its *B. cereus *ATCC 14579 parental strain. Two hours after the onset of the stationary phase, a stage at which the PlcR regulon is highly expressed, the *plcR *expression in the *yvfTU *mutant was only 50% of that of its parental strain. In addition to the reduced *plcR *expression in the *yvfTU *mutant, a few members of the PlcR regulon showed a differential expression, as revealed by transcriptomic and proteomic analyses. The virulence of the *yvfTU *mutant in a *Galleria mellonella *insect model was slightly lower than that of the parental strain.

**Conclusion:**

The YvfTU two-component system is not required for the expression of most of the virulence factors belonging to the PlcR regulon. However, YvfTU is involved in expression of *plcR*, a major regulator of virulence in *B. cereus*.

## Background

*Bacillus cereus *is a Gram-positive, rod-shaped, motile and spore-forming bacterium with opportunistic pathogen properties in human, causing local and systemic infections. *B. cereus *is mostly observed as a causative agent of food poisoning. This species belongs to the *B. cereus *group that includes the closely related species *Bacillus anthracis*, *Bacillus thuringiensis*, *Bacillus weihenstephanensis*, *Bacillus mycoide*s and *Bacillus pseudomycoide*s [1,2]. *B. cereus *produces several secreted proteins, including enterotoxins, cytolysins, phospholipases and proteases that may contribute to *B. cereus *pathogenicity. Expression of most of these virulence factors is controlled by the pleiotropic transcriptional activator PlcR [3-5]. This global regulator contributes to *B. cereus *virulence in mice, in insects [6] and to rabbit endophthalmitis [7]. Expression of the PlcR regulon starts at the onset of the stationary phase of growth [8]. It results from a cell-cell communication system that requires PapR, a peptide exported and re-imported in the bacterial cell as a mature form via an oligo-peptide permease [9,10]. The PlcR-PapR active complex binds a DNA target site, designated as the PlcR box, thus activating transcription of the PlcR regulon genes [3,10].

Bacteria recognize and respond to a variety of environmental stimuli using various signal transduction mechanisms, including two-component systems (TCS) [11]. TCS are characterized by a histidine kinase (HK) sensor coupled with a cognate response regulator. Perception of a particular stimulus by the HK leads to its autophosphorylation. The phosphoryl group is then transferred to the response regulator, usually leading to transcriptional activation of genes. TCS are widespread among bacteria, and the number of TCS encoding genes found in a bacterium is usually proportional to the size of its genome [12,13].

Genome sequencing of *B. cereus *strain ATCC 14579 has revealed the presence of 55 sensor kinases [14,15]. Most of them are also found in other members of the *B. cereus *group for which the genome sequence is available, including *B. anthracis *and *B. thuringiensis *[16].

TCS controlling expression of virulence factors have been shown in several bacteria, including *B. cereus*. Recently, the *B. cereus *ResDE TCS which is involved in low oxydo-reduction potential adaptation was shown to control enterotoxin production under anaerobiosis [17]. In contrast, the *B. cereus *YvrGH TCS, of which the encoding genes are located in the vicinity of the cytotoxin K encoding gene, was not required for expression of this virulence factor [18].

TCS encoding genes are often located in the same chromosomal region as the genes that they control, as for example in *B. subtilis *with the DesKR, CitST, YycFG, BceRS and LiaRS TCS [19-23]. In the *B. cereus *group, the TCS encoding *yvfTU *genes are located in the vicinity of *plcR*. To determine whether the YvfTU TCS plays a role in the expression of *plcR *and consequently of the PlcR regulon, the phenotype of a *yvfTU *mutant constructed in the *B. cereus *strain ATCC 14579 was analyzed.

## Results

### Identification of genes encoding a TCS in the vicinity of the *plcR *locus among the *B. cereus *group

**Figure 1:**
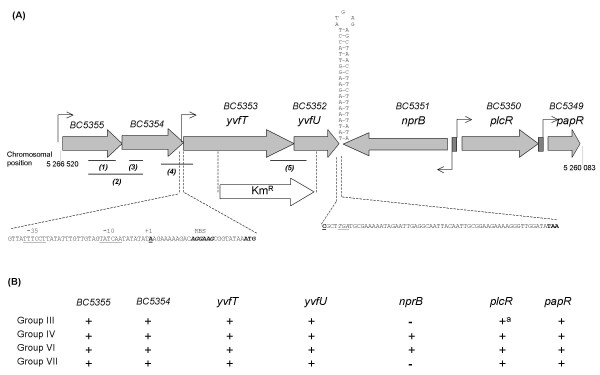
**The *B. cereus yvfTU *chromosomal region**. (A) Map of the  *B. cereus*  ATCC 14579  *yvfTU*  chromosomal region. Grey arrows represent ORFs. The position of the kanamycin resistance gene integrated in the chromosome of the mutant to disrupt *yvfTU *is indicated. Small arrows represent promoters or putative promoters. Function or putative function of gene products: BC5355, ABC transporter ATP-binding protein; BC5354, ABC transporter permease protein; *yvfT*, two-component sensor kinase; *yvfU*, two-component response regulator; *nprB*, metalloprotease; *plcR*, transcriptional activator; *papR*, PlcR activating peptide; Black box: PlcR recognition site. Lines annotated by numbers in brackets correspond to RT-PCR amplicons (see text and Fig. 2). In the *yvfT *promoter region, putative -10 and -35 sigma A boxes (underlined), initiation of transcription (bold underlined) and RBS (bold italic) are indicated In the *yvfU *3'-region, one cytosine (bold underlined) which leads to a stop codon (italic underlined) was indicated in the ATCC 14579 genome sequence, but was lacking in our sequence data which gives a different stop codon (bold). The predicted stem-loop at the end of *yvfU *is represented. (B) Synteny of the  *yvfTU*  chromosomal region among the  *B. cereus*  group. Taxonomic group determined as previously described [24]. Studied strains were: Group III, *B. cereus *ATCC 10987; *B. cereus *ZK (E33L); *B. thuringiensis *konkukian; *B. anthracis *Sterne; *B. anthracis *Ames; *B. anthracis *Ames 0581; *B. anthracis *Ames ancestor; *B. cereus *W, *B. thuringiensis *Al Hakam. Group IV, *B. cereus *ATCC 14579; *B. thuringiensis *israelensis ATCC 35646. Group VI, *B. weihenstephanensis *KBAB4. Group VII, *B. cereus *NVH 391/98, *B. cereus *NVH 883/00. ^a^: truncated in *B. anthracis *strains.

Genes encoding a TCS (BC5352 and BC5353) were located between 5,263 kb and 5,265 kb on the minus strand of the *B. cereus *ATCC 14579 chromosome (Fig. 1A). Using BlastP, the deduced amino acid sequences of BC5353 and BC5352 genes appeared to be homologous to both the *B. subtilis *YocF and YocG (now called DesK and DesR) (37 and 62% identity, with E-values of 2e-60 and 2e-42, respectively) and the *B. subtilis *YvfT and YvfU (43 and 62% identity, with E-values of 9e-78 and 4e-43, respectively). In *B. cereus *ATCC 14579, genes encoding a putative ABC transporter (BC5355 and BC5354) were located upstream from the BC5353 and BC5352 genes (Fig. 1A). Orthologous ABC transporter encoding genes were also located upstream from the *yvfTU *genes in *B. subtilis *(not shown). The BC5353 and BC5352 genes studied here have been annotated as *yocF *and *yocG *in the *B. cereus *strain ATCC 14579 [15]. However, these genes were previously annotated *yvfT *and *yvfU *in ATCC 14579 [5]. Because of (i) their higher sequence homologies with the *B. subtilis yvfT *and *yvfU *genes, (ii) their conserved synteny with the *B. subtilis yvfTU *locus (including ABC transporter encoding genes), and (iii) their previous annotations, the BC5353 and BC5352 genes were named as *yvfT *and *yvfU*, respectively, throughout the present work.

**Figure 2:**
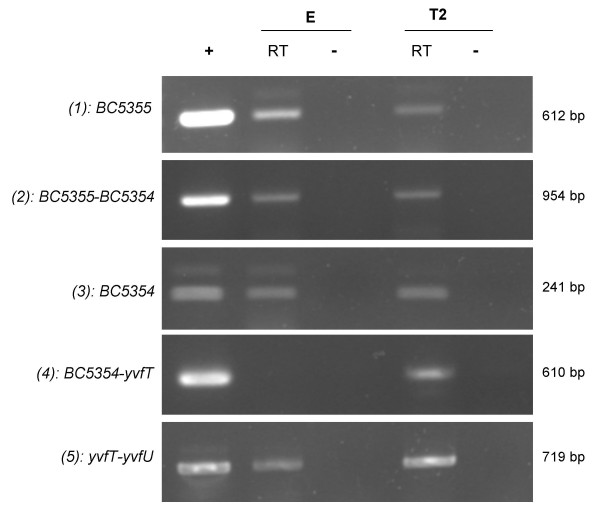
**RT-PCR detection of *yvfTU*, BC5354 and BC5355 in *B. cereus *strain ATCC 14579**. Lane "+": Positive control (PCR on genomic DNA). Lanes "RT": RT-PCR on 500 ng RNA. Lanes "-": Negative control (RT-PCR on 500 ng RNA with a heat-inactivated reverse-transcriptase). Numbers in brackets refer to the positions of the RT-PCR products on the *yvfTU *locus, as represented on Fig. 1A. RNA extraction was performed on strains grown at 37°C in LB broth and harvested in exponential phase (E) (OD_600 _1), or 2 hours after the onset of stationary phase (T2).

In the strain ATCC 14579, the *yvfTU *genes were located in the vicinity of *plcR *(BC5350) encoding a transcriptional regulator and its activating peptide encoding gene *papR *(BC5349) (Fig. 1A). A putative protease encoding gene, *nprB *(BC5351), was found between *yvfU *and *plcR*. Among the *B. cereus *group strains, the *yvfTU *genes were always located in the vicinity of *plcR-papR *(Fig. 1B). The synteny was conserved, except for *nprB *which is lacking in some strains of the *B. cereus *group. The presence or absence of the *nprB *gene seemed to be correlated to the taxonomic position of the strains [24,25], according to the genome sequences available up to now in each taxonomic group.

### Domains in YvfT and YvfU

Putative motifs present in the deduced amino-acid sequences of *yvfT *and *yvfU *were predicted according to the Smart tool: YvfT displays an ATP binding domain (HATPase domain), and a dimerisation and phosphoacceptor domain (PFAM:HisKA_3 domain). YvfT is most probably a membrane protein as revealed by the 4 predicted transmembrane domains in its N-terminal region. This histidine kinase belongs to the class IIa defined by Fabret *et al*. [26], or to the class 7 according to Grebe and Stock [27]. YvfU, the cognate response regulator of YvfT belongs to the NarL class of response regulators [27]. YvfU displays a phosphoacceptor site (REC domain) in the N-terminal region. The DNA-binding motif (HTH domain) found in the C-terminal region suggests a transcriptional regulator function. The YvfU TCS corresponds to the reference code 56 defined by de Been *et al*. [16].

According to the published genome sequence [15], the YvfU response regulator is smaller in strain ATCC 14579 than in other strains of the *B. cereus *group (not shown). However, our sequences from 3 independent PCR-amplified *yvfU *fragments differed from the genome data, by the absence of a cytosine at the position 420 in the *yvfU *gene which created a frameshift. The *yvfU *gene was consequently 55 bp longer (Fig. 1A), and the C-terminal-end of YvfU (which contains the HTH domain) included therefore 18 additional amino-acids. Thus, YvfU from the ATCC 14579 strain displays a higher similarity with the other *B. cereus *group members' YvfU protein (Additional file 1).

### Construction of a *yvfTU *mutant

A mutant was constructed in *B. cereus *ATCC 14579 by interrupting both the *yvfT *and *yvfU *genes. No difference was observed between Δ*yvfTU *and its parental strain during growth in LB at various incubation temperatures (15°C, 37°C and 42°C) (data not shown).

### Expression of genes mapping in the *yvfTU *locus

RT-PCR was performed on RNA extracted from ATCC 14579 cells grown either in exponential (OD_600 _= 1.0) or stationary phase at T2 (2 hours after the onset of stationary phase). The *yvfT *and *yvfU *genes were co-transcribed as indicated by the detection of mRNA overlapping both genes, during both exponential and stationary phase (panel 5 in Fig. 2). The genes encoding putative ABC transporters (BC5354 and BC5355, located just upstream from *yvfTU*) were also co-transcribed in the same growth conditions (panel 2 in Fig. 2). However, a signal overlapping BC5354 and *yvfT *genes was detected by RT-PCR only on RNA extracted from cells harvested at T2 (panel 4 in Fig. 2). Thus, at certain growth stages, these 4 genes could be co-transcribed as a single operon.

The co-transcription of the *yvfTU *genes alone (i.e. without a co-transcription with BC5355-BC5354) during exponential phase of growth suggested the existence of a specific promoter upstream from *yvfT*. This result was confirmed by the identification of the 5'-end of the *yvfTU *transcript mapping 25 bp upstream from the start codon (Fig. 1A), using the rapid amplification of cDNA ends (RACE)-PCR technique.

**Figure 3:**
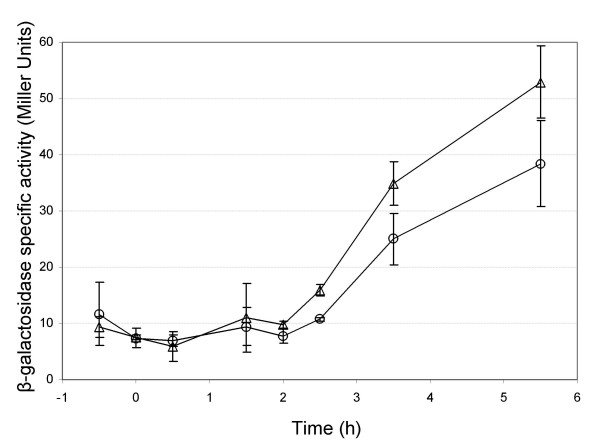
***yvfT *directed *lacZ *expression in *B. cereus *WT or *yvfTU *mutant strains**. β-galactosidase activity was measured in either the WT strain (circles) or in the *yvfTU *mutant (triangles) harbouring pHT-yvfT'Z (649 bp *yvfT *promoter region cloned upstream from the promoterless *lacZ *reporter gene in pHT304-18Z). Time 0 indicates the onset of the stationary phase of growth. Each curve is the mean value of triplicate measurements, representative of 3 independent experiments. Bars represent standard deviation.

A *yvfT*'*lacZ *transcriptional fusion was therefore constructed on a plasmid and transferred in both *B. cereus *ATCC 14579 and Δ*yvfTU *strains. Measurement of β-galactosidase activity throughout the kinetics of growth revealed a very low expression in both strains during the exponential phase and until two hours after the onset of stationary phase (mean values about 10 Miller units, Fig. 3). Then, β-galactosidase activity increased in both strains. These results indicate that *yvfTU *was transcribed from its own promoter at a basal level in exponential phase of growth and transcription was increased during the stationary phase. No significant difference between the *yvfT*-directed *lacZ *transcription in the 2 strains at any time of the growth culture was observed (*P *> 0.05, Student's *t *test).

Because RT-PCR experiments revealed a co-transcription of *yvfTU *with the two BC5355 and BC5354 genes at T2, quantification of the promoter activity of the BC5355-BC5354-*yvfT-yvfU *operon, was performed by constructing a BC5355'*lacZ *transcriptional fusion. Measurements of the β-galactosidase activity at T2 in the *B. cereus *ATCC 14579 and Δ*yvfTU *strains were (mean ± sd) 28 ± 4 and 22 ± 13 Miller Units, respectively, and were not significantly different (*P *> 0.05, Student t-test).

Thus, disruption of the chromosomal *yvfTU *genes did not alter the transcription of the plasmidic *yvfTU *promoter, nor that of the plasmidic BC5355 promoter, revealing that, in the tested conditions, the *yvfTU *operon was not autoregulated.

### *plcR *expression in the *yvfTU *mutant

**Figure 4:**
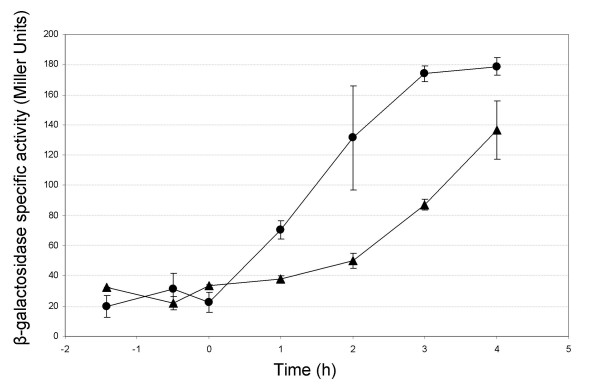
***plcR *directed *lacZ *expression in *B. cereus *WT or *yvfTU *mutant strains**. β-galactosidase activity was measured in either the WT strain (circles) or in the *yvfTU *mutant (triangles) harbouring pHT-plcR'Z (*plcR *promoter region cloned upstream from the promoterless *lacZ *reporter gene in pHT304-18Z). Time 0 indicates the onset of stationary phase. Each curve is the mean value of triplicate measurements, representative of 3 independent experiments. Bars represent standard deviation.

A transcriptional fusion between the *plcR*-promoter region from *B. cereus *strain ATCC 14579 and the *lacZ *reporter gene was constructed (Table 1) in order to determine the levels of *plcR *transcription in both the WT and Δ*yvfTU *strains throughout the kinetics of growth (Fig. 4). Measurement of β-galactosidase activity revealed that during the exponential phase, the *plcR *expression level was constant, ranging from 20 to 30 Miller units in both strains (no significant difference between the two strains). In contrast, during the stationary phase, *plcR *expression increased in both strains, but this increase was faster in the WT strain than in Δ*yvfTU*. Thus, during stationary phase, a higher *plcR *expression was detected in the WT strain as compared to that in Δ*yvfTU *(*P *< 0.05).

**Table 1:** Strains and plasmids used in this study

Strain or plasmid	Relevant genotype^a^	Source or reference
Strains		
*B. cereus *ATCC 14579		Laboratory collection
*B. cereus ΔplcR*	ATCC 14579 *plcR::Km*	[6]
*B. cereus ΔyvfTU*	ATCC 14579 *yvfTU::Km*	This work
*E. coli *TG1	[Δ(*lac-proAB*) *supE thi hsd*-5 (*F' traD36 proA*^+ ^*proB*^+ ^*lacI*^q ^*lacZΔ*M15)]	Laboratory collection
*E. coli *ET12567	(*F*^- ^*dam-13*::Tn*9 dcm-6 hsdM hsdR recF143 zjj-202*: :Tn*10 galK2 galT22 ara14 pacY1 xyl-5 leuB6 thi-1*)	Laboratory collection
		
Plasmids		
pUC18	Ap^R ^cloning vector	Laboratory collection
pMAD	Ap^R ^and Em^R ^shuttle vector	[45]
pHT304	Ap^R ^and Em^R ^cloning vector	[58]
pHT304-Km	*aphAIII *with its own promoter cloned in *Sal*I and *Pst*I sites of pHT304	This work
pMADΔ *yvfTU*	Recombinant pMAD harboring *yvfTU::Km*	This work
pHT304-18'Z	Ap^R ^and Em^R ^cloning vehicle; *lacZ *reporter gene	[48]
pHT-*yvfTU*	*yvfTU *and its promoter (2514 bp) cloned in *Pst*I and *Eco*RI sites of pHT304	This work
pHT-plcR'Z	1836 bp region upstream from *plcR *start codon cloned between the *Hind*III and *Xba*I sites of pHT304-18'Z	This work
pHT-yvfT'Z	649 bp region upstream from *yvfT *start codon cloned in *Pst*I and *Bam*HI sites of pHT304-18'Z	This work

^a ^Km, kanamycin; Ap, ampicillin; Em, erythromycin

### Expression of the PlcR regulon in the *yvfTU *mutant

To study the expression of the PlcR regulon in Δ*yvfTU*, in comparison with that of its parental strain, a transcriptomic approach was performed. Bacteria were grown in LB medium and were harvested two hours after the onset of the stationary phase of growth. At this time, 80% of the total amount of the extracellular proteins is produced in a PlcR-dependent manner [4].

The PlcR regulon has recently been thoroughly defined by a microarray-based approach [28]. Among the 45 genes belonging to this regulon, a significant differential expression of at least 1.5 fold was observed between the 2 strains for 11 of them by the transcriptome analysis (Table 2). These results revealed that the *yvfTU *mutation changed the expression of only a small part of the PlcR regulon at the transcriptional level, in the growth conditions used in this study.

**Table 2:** PlcR-regulon members differentially expressed in the *yvfTU *mutant

NCBI ID	Putative gene product	*yvfTU*/WT^b^	M-value^c^	*P*-value^d^	Putative localisation
BC0670	phosphatidylcholine-preferring phospholipase C (PC-PLC)	0.32	-1.649	0.004	Extracellular
BC0671^a^	sphingomyelinase C	0.33	-1.582	0.006	Extracellular
BC1809	enterotoxin (NheA)	0.53	-0.913	0.006	Extracellular
BC1810^a^	enterotoxin (NheB)	0.59	-0.757	0.034	Extracellular
BC3102^a^	Hemolysin BL binding component precursor (HblB)	1.94	0.953	0.027	Extracellular
BC3103^a^	Hemolysin BL lytic component L1 (Hbl-L1)	1.92	0.944	0.022	Extracellular
BC3104	Hemolysin BL lytic component L2 (Hbl-L2)	2.21	1.147	0.002	Extracellular
BC5101	thiol-activated cytolysin (Cereolysin O)	0.66	-0.610	0.029	Extracellular
BC5349	PapR protein	0.59	-0.773	0.033	Extracellular
BC5350	PlcR Transcriptional activator	0.52	-0.933	0.001	Cytoplasmic
BC5351	Bacillolysin (NprB)	0.34	-1.560	0.001	Extracellular

^a ^Genes without a PlcR box found directly upstream, but located in a putative PlcR-regulated operon.

^b ^Absolute ratio of expression in the *yvfTU *mutant vs WT. Up- or downregulation of more than 1.5 fold are presented.

^c ^Log_2 _of the absolute ratio. Negative M-value, downregulated in the mutant; positive M-value, upregulated in the mutant. M-values of more than 0.59 or less than -0.59 (corresponding to an 1.5 fold up- or downregulation in the mutant, respectively) are presented.

^d ^*P*-value < 0.05 obtained with the t-test was considered as significant

Eight of these genes showed a reduced expression in Δ*yvfTU*, including *plcR *and its activating peptide encoding gene *papR*. Relative real-time quantitative PCR (qRT-PCR) was performed and confirmed that in Δ*yvfTU*, the *plcR *mRNA levels represented 0.48 fold (mean of 3 independent experiments, range 0.31–0.60) of the mRNA levels in the WT. Some genes such as *nprB, nheAB, plcB*, encoding toxins or degradative enzymes, were also downregulated (Table 2). In contrast, the three genes encoding the haemolysin BL were overexpressed in the mutant. The *hblC *mRNA levels in qRT-PCR were 2.49 fold (range 2.39–2.60) higher in Δ*yvfTU *than in the WT and confirmed the results of the transciptomic analysis.

Complementation of the Δ*yvfTU *strain was performed by introducing the *yvfTU *genes under the control of their own promoter on a plasmid (Table 1). The *plcR *mRNA level was quantified in the resulting strain harvested 2 hours after the onset of stationary phase. In the complemented Δ*yvfTU *strain, the mRNA level of *plcR *was restored: it was 1.4 fold higher than in the WT strain and 1.7 fold higher than in the WT strain harbouring the control plasmid (pHT304).

Because most virulence factors belonging to the PlcR regulon are extracellular proteins, the extracellular proteome from 4 cultures of each of WT and Δ*yvfTU *strains was also analyzed. Identification of proteins on gels after a two-dimensional electrophoresis was based on previous spots annotations performed in similar experimental conditions [29]. For confirmation, 8 spots were analysed by Mass-Spectrometry.

**Figure 5:**
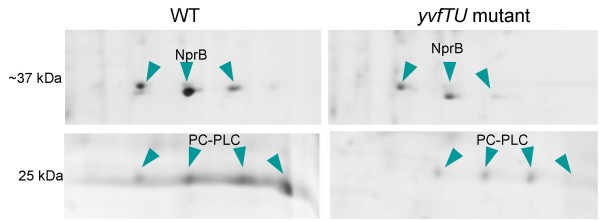
**2D-GE of WT and *yvfTU *mutant culture supernatants collected 2 hours after the onset of stationary phase**. Spots found in lower amount in the extracellular proteome of the *yvfTU *mutant correspond to NprB and PC-PLC proteins, as confirmed by Mass Spectrometry identification. The different spots correspond to isoforms of the same protein, with distinct charges but identical molecular weights. The gel areas shown are located around 37 kDa with a pI between 5.5 and 6 for NprB, and 25 kDa with a pI between 6.0 and 7.0 for PC-PLC.

Using the software ImageMaster Platinum, spot volume quantification was performed after normalization and a Kruskal-Wallis (KW) test was applied to detect reproducible differences between the extracellular proteomes of the two strains. Some differences were observed for spots corresponding to NprB and PC-PLC (Fig. 5). The three spots corresponding to three isoforms of NprB were found in a significant higher amount in the extracellular proteome of the WT (mean 7.09% vol, range 5.28–9.57) when compared to that of the *yvfTU *mutant (mean 3.31% vol, range 1.54–5.45) (differences in the KW test significant at *P *= 0.02). Similarly, the mean % vol of spots corresponding to PC-PLC (encoded by the *plcB *gene) represented 4.24 (range 3.22–6.16)% vol and 1.26 (range 0.16–2.89)% vol in the extracellular proteomes of the WT and Δ*yvfTU*, respectively (difference significant in KW test at *P *= 0.05).

The total amount of Nhe represented 8.21 (range 3.67–12.38)% vol and 5.20 (range 2.08–8.00)% vol (for NheA) and 6.14 (range 5.39–7.69) and 4.66 (range 3.59–7.24)% vol (for NheB), in the WT and Δ*yvfTU *extracellular proteomes, respectively. Although differences were not significant (*P *> 0.05 in KW test), in each of the 4 runs, the amount of NheA and NheB was always higher in the gels of the WT extracellular proteome than in those of Δ*yvfTU *(differences significant at *P *= 0.03 for NheB and at *P *= 0.06 for NheA in a paired t-test).

Apart from those differences, the extracellular proteomes of both strains were similar. In particular, the amount of the other extracellular proteins known to be expressed in a PlcR-dependent manner was not significantly affected by the *yvfTU *mutation, confirming the small number of changes in expression of the PlcR regulon observed by the transcriptomic analysis.

### Role of YvfTU in some virulence features

Virulence of the *yvfTU *mutant was estimated in a *Galleria mellonella *infection model. Injection of various doses (200 to 20,000 cfu) of WT and *yvfTU *mutant was performed into the *G. mellonella *hemocoel (blood of the insect).

By using a Probit analysis software, the LD_50 _at 24 hours post infection were estimated. For the wild type the LD_50 _was 2.5 × 10^3 ^(95% confidence limits from 1.8 × 10^3 ^– 3.6 × 10^3^) CFU and for the Δ*yvfTU *mutant the LD_50 _was 4.2 × 10^3 ^(95% confidence limits from 3.2 × 10^3 ^– 5.8 × 10^3^) CFU. Test of parallelism and the virulence ratios at the LD_50 _level showed a significant ((*P *< 0.05, X^2 ^test) but small decrease (1.6 fold with (1.3–2.1, 95% confidence limits)) in virulence with the Δ*yvfTU *mutant compared to the WT. No difference between Δ*yvfTU *and the WT in time-to-death was noticed (data not shown).

When grown on sheep blood agar, the Δ*yvfTU *and the WT strains displayed an identical hemolysis phenotype. The two strains did not show significant difference in cytotoxicity on HeLa cells and on macrophages (data not shown).

## Discussion

Some orthologs of the *B. cereus yvfTU *genes are found in other *Bacillus *species. However, in none of these organisms the YvfTU function has been studied. Although the exact function of many TCS is unknown, the TCS encoding genes are often located in the same chromosomal region as the genes that they control. Among the *B. cereus *group members, the *yvfTU *genes are highly conserved, and are always located in the vicinity of *plcR*. Because of this conserved synteny, we hypothesized a link between YvfTU and PlcR. Expression of *plcR *was therefore studied in a *yvfTU *mutant. The lower β-galactosidase activity measured in Δ*yvfTU *harboring a *plcR'lacZ *transcriptional fusion revealed a transcription of *plcR *partly depending on YvfTU. This dependence between YvfTU and *plcR *expression was confirmed by transcriptome analysis and quantitative real-time PCR.

Because the expression of *plcR *was affected by the *yvfTU *mutation, we wondered about a consequently modified expression of the PlcR regulon. The transcriptome and the extracellular proteome of both the WT and Δ*yvfTU *strains were analyzed, at the time of maximum protein concentration in the culture supernatant [30] and when 80% of the total amount of the produced proteins is expressed in a PlcR-dependent manner [4] (i.e. two hours after the onset of the stationary phase). At this stage, *yvfTU *genes were transcribed, and the *plcR *transcription was partly YvfTU-dependent. Despite a lower level of *plcR *mRNA in Δ*yvfTU*, the transcriptomic and proteomic analysis both revealed that the expression of the PlcR regulon was mostly not affected by the *yvfTU *mutation. This suggests that PlcR can play its regulatory role even when expressed at a low level.

However, our results revealed that the decrease in *plcR *mRNA levels caused by the *yvfTU *mutation was concomitant to a differential expression at the transcriptional level of 11 PlcR-regulated genes, with 8 genes showing a reduced expression in Δ*yvfTU*. An increase in expression of the 3 genes belonging to the *hbl *operon was observed in Δ*yvfTU *where *plcR *is expressed at a lower level, while *hbl *is known to be expressed in a PlcR-dependent manner [3,4]. Although performed in different growth conditions, such a lack of congruence between the trends of transcription of several PlcR-regulated genes (including *hbl*) and expression of *plcR *itself has been previously shown [31]. Furthermore, the recent discovery of the involvement of another regulator (Fnr) in *hbl *expression emphasizes the fine regulations occurring in *B. cereus *in addition to the PlcR regulation [32,33]. The differential response of several genes among a particular regulon has been observed many times elsewhere, and it was recently suggested that in *B. cereus*, some diversity in the regulation of gene expression occurs within the PlcR regulon [30]. All these findings, coupled with our results, suggest that expression of *hbl *and some other members of the PlcR regulon can undergo subtle regulations which also involve yet unidentified factors, different from PlcR.

For several genes showing reduced expression at the transcriptional level in Δ*yvfTU*, a concomitant decrease in the amount of their products was detected by 2D gel analysis. In some cases, differential expression was only observed with the transcriptomic approach (for example, the Cereolysin O encoding gene). The relatively low levels of differential expression observed with the transcriptome analysis suggest that the proteomic approach was less sensitive in our conditions. Furthermore, various post-transcriptional regulations might explain the lack of correlation between transcriptomic and proteomic approaches, as reported in several studies on other organisms (for example, see references [34-37]).

In bacterial pathogens, several TCS mutants present attenuation in virulence [38]. In our study, the virulence of Δ*yvfTU *in the *G. mellonella *insect was also slightly lower than that of the WT strain. The reduced expression of *nprB*, *plcB *and *nheAB *coupled with the lower amount of their products found in the extracellular proteome of Δ*yvfTU *may account for this reduction in virulence. However, this only slightly impaired virulence is not surprising, as Δ*yvfTU *did not present a major alteration of the PlcR regulon, and considering the importance of this regulon in *B. cereus *insect virulence [6].

Expression of chromosomal genes not regulated by *plcR *was also affected by the *yvfTU *mutation, as observed with the transcriptomic analysis (additional file 2). Among them, 29 and 25 genes showed respectively higher and reduced expression by 2 fold or more. However, no major pathway was over-represented in this gene list, giving no clue for the identification of a signal triggering the activation of the YvfTU TCS. An alignment of 1,000 bp promoter regions of the genes listed in additional file 2 was performed using the MEME programme. However, no conserved sequence was identified, suggesting that no highly conserved regulatory motif was responsible for an YvfTU-dependent differential expression in these promoter regions.

## Conclusion

This work showed that YvfTU and PlcR are genetically linked. A functional link was also shown by the identification of a YvfTU dependent *plcR *expression. The decreased *plcR *expression observed in Δ*yvfTU *only slightly modified the expression of the PlcR regulon, and slightly decreased the virulence of *B. cereus *in an insect model. A subtle regulation for some virulence factors produced by *B. cereus *exists in addition to the PlcR regulation.

## Methods

### Strains and growth conditions

All bacterial strains and plasmids used in this study are listed in Table 1. *E. coli *and *B. cereus *cells were routinely grown in Luria broth (LB) medium with vigorous agitation at 37°C. When required, the antibiotic concentrations used for bacterial selection were: erythromycin at 10 μg ml^-1 ^or kanamycin at 150 μg ml^-1 ^for *B. cereus *and ampicillin at 100 μg ml^-1 ^for *E. coli*. Bacteria with the Lac^+ ^phenotype were identified on LB agar containing 40 μg ml^-1^X-Gal. Columbia agar plates containing 5% sheep blood (BioMérieux) were used to assay the hemolytic activity of the strains.

### In silico analysis

tblastn alignments were performed on NCBI web site: [39]. Protein domains in YvfT and YvfU were identified using SMART software [40,41]. MEME programme was used to perform alignments of promoters of upregulated and downregulated genes in the *yvfTU *mutant [42].

### DNA manipulation

Plasmid DNA was extracted from *E. coli *and *B. cereus *by a standard alkaline lysis procedure using the Wizard SV miniprep purification system (Promega), with the following modification in the first step of the lysis procedure for *B. cereus*: incubation at 37°C for 1 h with 5 mg of lyzosyme (14,300 U mg^-1^). Chromosomal DNA was extracted from *B. cereus *cells harvested in mid-log phase as described previously [43]. Restriction enzymes and T4 DNA ligase were used as recommended by the manufacturer (Promega). Oligonucleotide primers were synthesized by Eurogentec. PCR was performed in a GeneAmp PCR system 2400 thermal cycler (Perkin-Elmer), using the Expand high fidelity DNA polymerase (Roche). Amplified DNA fragments were purified by using the PCR purification Kit (Roche) and separated on 0.7% agarose gels after digestion. Digested DNA fragments were extracted from agarose gels with a centrifugal filter device (montage DNA gel extraction kit; Millipore). All constructions were confirmed by DNA sequencing (GenomeExpress, Grenoble, France). Electroporation to transform *B. cereus *was used as previously described [44].

### RT-PCR and characterization of the *yvfT-yvfU *transcriptional unit

Total RNA was extracted from *B*. *cereus *ATCC 14579 wild-type (WT) cells grown aerobically in LB medium at the end of exponential phase (OD_600 _= 1.0), or two hours after the onset of stationary phase (T2), using the RNA extraction Pro-blue kit as recommended by the manufacturer (Q-Biogen). cDNA synthesis from 1 μg of total RNA was performed by using AMV-RT polymerase according to the instructions given by the RT-PCR kit (Roche). Specific amplifications were performed with the primers 5355-F and 5355-R (Table 3) for the BC5355 gene, 5355-F and 5355-54-R for a region overlapping BC5355 and BC5354, PyvfT-F and 5354-R for the BC5354 gene, 5Up-KR and yvfT-54-R for a region overlapping BC5354 and *yvfT*, and yvfTU-F and yvfTU-R for a region overlapping *yvfT *and *yvfU*. This step was coupled with 30 cycles of PCR amplification with Expand-HighFidelity polymerase as recommended (Roche). The *yvfTU *transcription start site was determined by the RACE-PCR kit (Roche) using yvfT-R2 and yvfT-R3 oligonucleotides (Table 3), following the manufacturer's instructions.

**Table 3:** Primers used in this study

Primer name	5'-3' sequence^a^	Restriction sites
5Up-KR	GCTACCATGGCTTCTAAAAATTACACCGCTTC	*Nco*I
3Up-KR	GCTAGTCGACCATTAAAATAAACATTGGAACG	*Sal*I
5Dn-KR	GCTACTGCAGAGATTTAATGTTTGGCTTATGG	*Pst*I
3Dn-KR	GGTAGGATCCTACACGGTTTTGATTCTACTGA	*Bam*HI
Km5in	TCTGGTCGACCATTTGAGGTGATAGG	*Sal*I
Km3in	GCTACTGCAGATCGATACAAATTCCTCGTAGGCG	*Pst*I
Km5out	CGGTATAATCTTACCTATCACC	
Km3out	TACTCTGATGTTTTATATCTTTTCTAA	
PyvfT-F	CGCCTGCAGTTATTAATGCCTGTTATGTTTT	*Pst*I
PyvfT-R	CGCGGATCCTGTCTTTTTCTTATATATATTG	*Bam*HI
Cp-yvfTU	CGTCGAATTCATTTGAAGCACACGGTTAC	*Eco*RI
PplcR-R	GGCGTCTAGACCCATTAGAACAATCTAATTTT	*Xba*I
PplcR-F	GACGAAGCTTATTCATTTGATACGGCAGTG	*Hind*III
5355-F	GATAGTATTACGGTGGAAGAGG	
5355-R	AAACTGTTCAAATGCTTCAT	
5355-54-R	CTGTTTGAGCGATAATTTTT	
5354-R	CTGTTTGAGCGATAATTTTT	
yvfT-54-R	CTATTACAAGCTTCCATCCTGATGCC	
yvfTU-F	TTGTGAAAAATCCAGAGCGTGC	
yvfTU-R	ATCCAATCCACTTTGAATCGGC	
yvfT-R2	CTATTACAAGCTTCCATCCTGATGCC	
yvfT-R3	CGCAAGCTTCAAACCACATATACGGGAAG	
LC-16S-F	GGTAGTCCACGCCGTAAACG	
LC-16S-R	GACAACCATGCACCACCTG	
LC-plcR-F	TCCAGCAATTTCTTCAATGG	
LC-plcR-R	TCGGCATGATATTTCAATCG	
LC-hblC-F	TTCAAGCAGAAACTCAACAG	
LC-hblC-R	TTCAAGTCTATCCGAAAACC	

^a ^Restriction enzyme sites are underlined

### Mutant construction

The two contiguous genes encoding the TCS YvfTU were interrupted by allelic exchange with a cassette conferring kanamycin resistance (KmR) in *B. cereus *ATCC 14579 as previously described [45]. Briefly, a fragment of 912 bp corresponding to the upstream region of BC5353 (*yvfT*) was PCR amplified using primers 5Up-KR and 3Up-KR (Table 3). Similarly, a 967 bp fragment corresponding to the downstream region of BC5352 (*yvfU*) was PCR amplified using primers 5Dn-KR and 3Dn-KR (Table 3). PCR fragments were cloned in the pUC18 plasmid, in accordance with the endonuclease restriction sites previously introduced in the primers (Table 3). In parallel, the 1.5 kb fragment corresponding to the *aphA3 *kanamycin resistance gene with its own promoter was PCR amplified using primers Km5in and Km3in, and pDG783 as a DNA template [46,47], and cloned in the pHT304 plasmid (Table 1). The DNA fragments corresponding to the upstream and downstream *yvfTU *region and the KmR cassette were digested with the appropriate enzymes, purified and cloned altogether in the *Nco*I and *Bam*HI sites of the thermosensitive plasmid pMAD (Table 1). Ten μg of the recombinant plasmid pMADΔ*yvfTU *were used to transform *B. cereus *ATCC 14579, and subjected to allelic exchange as previously described [45]. Strains that were resistant to kanamycin and sensitive to erythromycin arose through a double cross-over event in which the chromosomal *yvfTU *copy was replaced with the KmR cassette. The chromosomal allele exchange in the *yvfTU *mutants was checked by DNA sequencing of PCR fragments amplified using the primers couples Km5out/5Up-KR and Km3out/3Dn-KR.

Complementation of the mutant was performed as follows: a PCR amplified fragment using primers PyvfT-F and Cp-yvfTU was cloned between the *Pst*I and *Eco*RI sites of pHT304 and introduced in *B. cereus *Δ*yvfTU *by electroporation.

### Construction of *lacZ *transcriptional fusions and β-Galactosidase assay

The DNA fragment harbouring the promoter regions were PCR amplified and digested according to the endonuclease sites introduced in the primers (see Table 3). The *lacZ *transcriptional fusions were constructed by cloning these DNA fragments between the corresponding sites of the low copy plasmid pHT304-18'Z [48]. The recombinant plasmids were introduced into *B. cereus *ATCC 14579 WT and Δ*yvfTU *strains by electroporation. *B. cereus *strains harbouring plasmids with *lacZ *transcriptional fusions were cultivated in LB medium at 37°C. β-Galactosidase specific activities were measured in triplicate samples from each culture as previously described [43] and were expressed in units of β-galactosidase per milligram of protein (Miller units). Total proteins in the sample were quantified using the Bradford method (BioRad protein assay). Experiments were repeated three times. Error bars indicate standard deviations of triplicate measurements.

### Transcriptome analysis

Three independent cultures of *B. cereus *ATCC 14579 WT and Δ*yvfTU *strains were cultured in LB medium at 30°C with shaking and harvested 2 hours after the onset of the stationary phase of growth (T2), as described previously [4].

Total RNA was extracted with the protocol described previously [31] modified as follows: frozen cell pellets from 10 ml culture were supplemented with 1 ml of TES buffer (0.03 M Tris pH 8.0, 0.005 M EDTA, 0.05 M NaCl). Disruption of the cells was performed by adding 0.3 g sterile glass-beads (Sigma) followed by a 40 sec. run on a FastPrep Instrument (MP Biomedical). For each slide, cDNA was generated as described previously on 20 μg of isolated RNA from the wild-type strain and 20 μg of RNA from the mutant strain [31]. After purification, the cDNA's were labeled with Cy3 (wild-type) and Cy5 (mutant) and vice versa for the dye-swapped slides. The combined cDNA's of wild-type and mutant were hybridized onto a micro-array of 70-mer oligonucleotides, which represent all 5,255 open reading frames of the *B. cereus *ATCC 14579 genome (design: see [31]). As described previously [31], the slides were incubated overnight at 42°C and, after washing, scanned with an Axon 4000B scanner (Molecular Devices Corp., California). Further processing of the scanned slide and the subsequent data analysis was performed as described [31], using GenePix Pro, version 6.0 software (Molecular Devices Corp.).

### Relative quantification of gene expression by real-time PCR

Real-time RT-PCR was performed on a Light-Cycler instrument (Roche) as previously described [17]. Briefly, the LightCycler RNA Amplification kit SYBR Green I (Roche) was used according to the manufacturer's instructions, with the following modifications: 5 ng of total RNA were used as a template; the reverse transcription step was performed at 50°C, and the annealing temperature during the 45 cycles of amplification was 50°C. Oligonucleotides listed in Table 3 with a name beginning with "LC" were used for Real-time PCR. PCR amplification were performed on 500 ng of each RNA sample, in order to check for absence of contaminating DNA. The mRNA level changes for each gene were normalized to the RNA level of the *ssu *gene encoding 16S RNA and quantified as previously described [49].

### Extracellular proteome analysis

Independent cultures of WT (n = 4) and Δ*yvfTU *(n = 4) were performed as indicated above for transcriptome analysis. As previously described [4], the supernatant of harvested cultures was filter-sterilized and proteins were precipitated with deoxycholate-tetrachloroacetic acid method. After ethanol:ether (vol/vol) washing, the protein pellet was stored at -80°C until use. Quantification of the protein content in the samples was performed by the Bradford method. For both strains, 100 μg of total proteins were loaded on an immobilized pH gradient strip (pH 4 to 7, 17 cm length, BioRad). After the isoelectro-focalisation, the strips were loaded on 12% polyacrylamide gels before the second dimension was run. Gels were silver stained [50], scanned, and analyzed with the ImageMaster platinum software (Amersham Biosciences) using the total spot volume normalization procedure. Four electrophoresis runs were performed with two gels, one of each WT and one of each Δ*yvfTU *supernatants. Finally, 8 gels were analyzed.

Statistical analysis of relative spot quantification was performed as follows: comparison of spot relative volumes in 4 replicate cultures of the extracellular proteomes of both strains was carried out using Kruskall Wallis non parametric test. A paired t-test was used to detect differences in spots volumes between strains in each of the 4 different runs (Systat version 9; SPSS; Chicago, IL, USA).

When necessary, a Coomassie-Blue staining [51,52] was performed on gels loaded with 500 μg of proteins, and spots were excised from the gels and digested with 0.1 to 0.5 μg of trypsin (Promega) at 37°C during 6 h. The digested proteins were analysed by MALDI-TOF MS on the PAPSS (Plateau d'Analyse Protéomique par Séquençage et Spectrométrie de Masse) at INRA Jouy-en-Josas, France. Peptide mass fingerprints were analyzed with ProteinProspector and Mascott softwares using the *B. cereus *ATCC 14579 genome database [53].

### Cytotoxicity of the *yvfTU *mutant

Cytotoxicity assay was performed on HeLa cells and murine macrophages as described previously [43,54].

### Insects and infection experiments

The virulence-related properties of *yvfTU *were assessed by comparing the killing effect of the *B. cereus *WT and Δ*yvfTU *strains by infection in 5^th ^instar (last larval stage before pupation) *Galleria mellonella *larvae. *G. melonella *eggs were hatched at 25°C and the larvae reared on beeswax and pollen (Naturalim). Groups of 20 *G. mellonella *larvae, each weighing about 200 mg, were used. Direct injection into the hemocoel was performed with various doses (ranging from 200 to 20,000 cfu) of vegetative bacteria, collected during exponential growth (OD_600 _= 1). Tests were run 5 times with 4–5 different doses per test.

Infected larvae were kept at 37°C and mortality was recorded at 24 H and 48 h. The 50% lethal doses (LD_50s_) values were estimated by Probit analysis at 24 H post infection [55,56]. The Probit program (Praxeme, France) tests for the linearity of dose-mortality curves, provides lethal doses and the slope of each dose-mortality line. It tests the parallelism of 2 or more dose-mortality lines and determines the virulence ratio between the bacterial strains. Using the X^2 ^(Chi^2^) test, the ratio is considered to be significantly different (*P *< 0.05) when the confidence limits do not include the value 1.

## Authors' contributions

JB designed the study, performed the majority of the experiments, analysed the data, and wrote the manuscript. KS performed the transcriptome experiments, CM performed the proteome experiments, CD performed the phenotypic characterisation of the mutant, MG participated in the design of the proteome experiments, CNL analysed the data of the insect virulence experiments and contributed to writing, NR did cytotoxicity assays. ABK, CNT, DL and VB participated in the design of the study. VB participated in the analysis of the data. All authors read, critically revised, and approved the final manuscript.

## Supplementary Material

Additional File 1 **Alignments between the C-terminal ends of YvfU from members of the *B. cereus *group**. Alignments were performed with the MultAlin version 5.4.1 program [57]. The YvfU sequences were deduced from the genome sequences available in the databases. Diverging amino acids are shown in grey boxes. The HTH domain is double underlined. Ba: *B. anthracis*. The YvfU sequence of strains Ames Ancestor, Ames and Sterne is similar and is indicated only once. Btkonk: *B. thuringiensis *serovar *konkukian *str. 97-27; BtAlHakam: *B. thuringiensis *str. *Al Hakam*; Bc10987: *B. cereus *ATCC10987; BcZK: *B. cereus *EL33; BcG9241: *B. cereus *G9241; BwKBAB4: *B. weihenstephanensis *KBAB4; Bc39198: *B. cereus *subsp *cytotoxicus *strain NVH 391-98; Bc14579: *B. cereus *ATCC 14579 (genome data); Bc14579cor: *B. cereus *ATCC 14579 with the corrected *yvfU *sequence (according to our sequence data). Click here for file

Additional File 2 **Genes 2 fold differentially expressed in the *B. cereus yvfTU *mutant strain as identified by transcriptome analysis.** Click here for file
