# Supplementary material for: The YvfTU Two-component System is involved in plcR expression in Bacillus cereus
Source: BMC Microbiol. 2008 Oct 16;8:183. doi: 10.1186/1471-2180-8-183 (PMC2588459; doi:10.1186/1471-2180-8-183)
Supplement: Additional File 1 — Alignments between the C-terminal ends of YvfU from members of the B. cereus group. Alignments were performed with the MultAlin version 5.4.1 program [57]. The YvfU sequences were deduced from the genome sequences available in the databases. Diverging amino acids are shown in grey boxes. The HTH domain is double underlined. Ba: B. anthracis. The YvfU sequence of strains Ames Ancestor, Ames and Sterne is similar and is indicated only once. Btkonk: B. thuringiensis serovar konkukian str. 97-27; BtAlHakam: B. thuringiensis str. Al Hakam; Bc10987: B. cereus ATCC10987; BcZK: B. cereus EL33; BcG9241: B. cereus G9241; BwKBAB4: B. weihenstephanensis KBAB4; Bc39198: B. cereus subsp cytotoxicus strain NVH 391-98; Bc14579: B. cereus ATCC 14579 (genome data); Bc14579cor: B. cereus ATCC 14579 with the corrected yvfU sequence (according to our sequence data). [file 1471-2180-8-183-S1.doc]

Ba FGLWQEQNPL SDREKEVLLL AKEGKTANEI AKALYLSPGT VRNYISEVLT KLDAKNRIEA ITIAEEKGWI

Btkonk FGLWQEQNPL SDREKEVLLL AKEGKTANEI AKALYLSPGT VRNYISEVLT KLDAKNRIEA ITIAEEKGWI

BtAlHakam FGLWQEQNPL SDREKEVLLL AKEGKTANEI AKALYLSPGT VRNYISEVLT KLDAKNRIEA ITIAEEKGWI

Bc10987 FGLWQEQNPL SDREKEVLLL AKEGKTANEI AKALYLSPGT VRNYISEVLT KLDAKNRIEA ITIAEEKGWI

BcZK FGLWQEQNPL SDREKEVLLL AKEGKTANEI AKALYLSPGT VRNYISEVLT KLDAKNRIEA ITIAEEKGWI

BcG9241 FGLWQEQNPL SDREKEVLLL AKEGKTANEI AKALYLSPGT VRNYISEVLT KLDAKNRIEA ITIAEEKGWI

BwKBAB4 FGLWQEQNPL SDREKEVLLL AKEGKTANEI AKALYLSPGT VRNYISEVLT KLDAKNRIEA ITIAEEKGWM

Bc39198 FGLWQEQNPL SDREREVLLL AKEGKTTNDI AKALYLSPGT VRNYISEVLT KLNAKNRIEA ITIAEEKGWI

Bc14579 FGLWQEQNPL SDREKEVLLL AKEGKTANEI AKALYLSPGT VRNYISEVLA NA

Bc14579cor FGLWQEQNPL SDREKEVLLL AKEGKTANEI AKALYLSPGT VRNYISEVLA KLDAKNRIEA ITIAEEKGWI
