# Supplementary material for: The YvfTU Two-component System is involved in plcR expression in Bacillus cereus
Source: BMC Microbiol. 2008 Oct 16;8:183. doi: 10.1186/1471-2180-8-183 (PMC2588459; doi:10.1186/1471-2180-8-183)

Chromosomal genes up- or downregulated 2 fold in *ΔyfTU*

ID	Name	M-value[a]	TIGR categorie
BC0248	hypothetical protein	4.252	hypothetical proteins
BC0250	hypothetical protein	4.436	hypothetical proteins
BC0251	hypothetical protein	3.237	hypothetical proteins
BC0294	chaperonin. 10 kDa	1.176	protein fate
BC0670	phospholipase C	-1.649	fatty acid and phospholipid metabolism
BC0671	sphingomyelinase C	-1.582	fatty acid and phospholipid metabolism
BC0841	glucokinase regulator-related protein	1.148	unknown function
BC0939	Type II restriction-modification system restriction subunit	-1.707	DNA metabolism
BC0940	Type II restriction-modification system restriction subunit	-1.649	DNA metabolism
BC0941	Type II restriction-modification system methylation subunit	-1.781	DNA metabolism
BC1009	hypothetical protein	1.882	hypothetical proteins
BC1431	Cell wall endopeptidase. family M23/M37	1.114	protein fate
BC1538	hypothetical protein	1.416	unknown function
BC1540	3-methyl-2-oxobutanoate hydroxymethyltransferase	1.497	biosynthesis
BC1541	pantoate--beta-alanine ligase	1.504	biosynthesis
BC1542	aspartate 1-decarboxylase	1.412	biosynthesis
BC1807	Amino acid permease	-1.207	cell envelope
BC1953	NLP/P60 family protein	1.070	cellular processes
BC2056	conserved hypothetical protein	-1.021	hypothetical proteins
BC2300	oxalate:formate antiporter. putative	2.452	transport and binding proteins
BC2428	hypothetical protein	1.523	hypothetical proteins
BC2776	dihydrolipoamide dehydrogenase	-1.604	energy metabolism
BC2777	dihydrolipoamide acetyltransferase	-1.416	energy metabolism
BC2778	TPP-dependent acetoin dehydrogenase E1 beta-subunit	-1.701	energy metabolism
BC2779	TPP-dependent acetoin dehydrogenase E1 alpha-subunit	-1.657	energy metabolism
BC3063	pyrrolidone-carboxylate peptidase	1.229	protein fate
BC3066	conserved hypothetical protein	1.047	unknown function
BC3068	conserved hypothetical protein TIGR00370	1.105	hypothetical proteins
BC3069	transcriptional regulator. IclR family	1.190	regulatory functions
BC3101	Hemolysin BL binding component precursor	1.078	cellular processes
BC3104	Hemolysin BL lytic component L2	1.147	cellular processes
BC3694	N-acetylmuramoyl-L-alanine amidase	-1.849	cell envelope
BC3698	Cell wall endopeptidase. family M23/M37	-2.890	protein fate
BC3699	Antigen	-2.798	cell envelope
BC3706	transcriptional repressor GlnR	1.829	regulatory functions
BC4087	phosphopentomutase	1.011	purines, pyrimidines, nucleosides and nucleotides
BC4313	GrpE protein	1.294	protein fate
BC4792	cytochrome d ubiquinol oxidase. subunit I	-1.457	energy metabolism
BC4793	cytochrome d ubiquinol oxidase. subunit II	-1.099	energy metabolism
BC4870	L-lactate dehydrogenase	1.893	energy metabolism
BC5062	tyrosyl-tRNA synthetase	1.209	protein synthesis
BC5116	hypothetical protein	-1.032	hypothetical proteins
BC5117	ABC transporter permease protein	-1.421	transport and binding proteins
BC5118	ABC transporter ATP-binding protein	-1.168	transport and binding proteins
BC5119	hypothetical protein	-1.563	hypothetical proteins
BC5120	hypothetical Cytosolic Protein	-1.552	hypothetical proteins
BC5121	hypothetical protein	-1.239	hypothetical proteins
BC5122	hypothetical Cytosolic Protein	-1.267	hypothetical proteins
BC5123	hypothetical protein	-1.210	hypothetical proteins
BC5186	cell division ABC transporter. ATP-binding protein FtsE	1.283	transport and binding proteins
BC5239	conserved domain protein	1.500	cellular processes
BC5351	Bacillolysin	-1.560	protein fate
BC5357	Collagen adhesion protein	1.022	protein fate
BC5438	membrane protein. putative	1.206	unknown function

[a]M-value, as described in Table 4

**categories**	total	up	Dn
hypothetical proteins/unknown fuctions	17	10	7
transport and binding proteins	4	2	2
energy metabolism	7	1	6
regulatory functions	2	2	0
biosynthesis	3	3	0
cellular processes	4	4	0
protein fate	7	5	2
purines, pyrimidines	1	1	0
protein synthesis	1	1	0
cell envelope	3	0	3
DNA metabolism	3	0	3
fatty acid and phospholipid metabolism	2	0	2
total	54	29	25

**broader categories** (up-/downregulated)	
hypothetical proteins (10/7)	17
metabolism (1/11)	12
protein fate (5/2)	7
cellular processes (4/0)	4
transport and binding proteins (2/2)	4
cell envelope (0/3)	3
biosynthesis (3/0)	3
regulatory functions (2/0)	2
protein synthesis (1/0)	1
other (1/0)	1
total	54

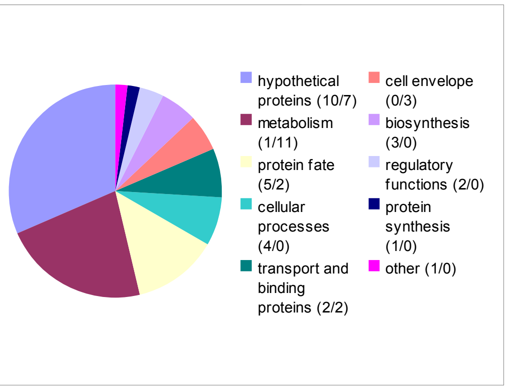

Supplement: Additional File 2 — Genes 2 fold differentially expressed in the B. cereus yvfTU mutant strain as identified by transcriptome analysis. [file 1471-2180-8-183-S2.pdf]
